# The 3D Collagen Network as a Determinant of Tumor Progression and Drug Delivery Efficiency in Breast Cancer

**DOI:** 10.3390/pharmaceutics18030340

**Published:** 2026-03-10

**Authors:** Mariana Hirata, Rogerio Padovan Gonçalves, Maria Eduarda Teixeira Pereira Cândido da Silva, Geovanna de Castro Feitosa, Caio Sérgio Galina Spilla, Domingos Donizeti Roque, Lisete Horn Belon Fernandes, Virgínia Maria Cavallari Strozze Catharin, Vitor Cavallari Strozze Catharin, Leila Maria Guissoni Campos, Ana Luiza Decanini Miranda de Souza, Eliana de Souza Bastos Mazuqueli Pereira, Juliana da Silva Soares de Souza, Maricelma da Silva Soares de Souza, Paulo Cezar Novais, Júlia Carolina Ferreira, Rose Eli Grassi Rici, Karina Torres Pomini

**Affiliations:** 1 School of Medicine, University of Marília (UNIMAR), Avenida Hygino Muzzy Filho, 1001, Marília 17525-902, São Paulo, Brazil; 2 Interdisciplinary Pos Graduate Program in Structural and Functional Interactions in Rehabilitation, University of Marília (UNIMAR), Avenida Hygino Muzzy Filho, 1001, Marília 17525-902, São Paulo, Brazil; 3 Department of Human Morphophysiology, School of Medicine, University of Marília (UNIMAR), Avenida Hygino Muzzy Filho, 1001, Marília 17525-902, São Paulo, Brazil; 4 Theoretical Bases of Mental Health, School of Medicine, University of Marília (UNIMAR), Avenida Hygino Muzzy Filho, 1001, Marília 17525-902, São Paulo, Brazil; 5 Department of Biochemistry and Pharmacology, School of Medicine, University of Marília (UNIMAR) Avenida Hygino Muzzy Filho, 1001, Marília 17525-902, São Paulo, Brazil; 6 School of Physiotherapy, University of Marília (UNIMAR), Avenida Hygino Muzzy Filho, 1001, Marília 17525-902, São Paulo, Brazil; 7 Department of Prosthodontics, University of Marília (UNIMAR), Avenida Hygino Muzzy Filho, 1001, Marília 17525-902, São Paulo, Brazil; 8 Faculty of Veterinary Medicine, University of Marília (UNIMAR), Avenida Hygino Muzzy Filho, 1001, Marília 17525-902, São Paulo, Brazil; 9 Department of Periodontics, School of Medicine, University of Marília (UNIMAR), Avenida Hygino Muzzy Filho, Marília 17525-902, São Paulo, Brazil; 10 Graduate Program in Anatomy of Domestic and Wild Animals, University of São Paulo (USP), São Paulo 05508-270, São Paulo, Brazil

**Keywords:** breast neoplasm, adenocarcinoma, extracellular matrix, collagen, tumor microenvironment

## Abstract

**Background/Objectives:** Breast cancer is a biologically complex malignancy whose high prevalence and therapeutic resistance represent a continuous challenge for global health. The Tumor Microenvironment (TME) is a crucial component in disease progression, and the Extracellular Matrix (ECM), particularly its 3D collagen architecture, is recognized for mediating interactions that influence invasion, metastasis, and pharmacological response. This review aims to critically synthesize recent evidence to elucidate the multifaceted role of collagen in the progression and modulation of therapeutic response in breast adenocarcinoma. **Methods:** A comprehensive literature review was conducted, analyzing studies addressing specific collagen subtypes, ECM stiffening (fibrosis), biomechanical signaling, and their impact on drug transport kinetics and immunomodulatory effects. **Results:** The results demonstrate that structural alterations of collagen not only orchestrate a pro-tumoral microenvironment, fostering aggressive phenotypes and immune evasion, but also create a physical barrier that compromises drug delivery efficiency and promotes metastatic dissemination. The synthesis of the data reinforces collagen as a potent prognostic biomarker and a promising therapeutic target for overcoming stroma-mediated resistance. **Conclusions:** Targeting the collagen-rich stroma and its 3D network is a critical frontier for therapeutic innovation. Developing adjuvant strategies to modulate the ECM has the potential to enhance clinical outcomes and optimize the distribution of antineoplastic agents, especially in patients with high degrees of tumor fibrosis.

## 1. Introduction

Breast cancer remains the most prevalent malignant neoplasm among women and one of the leading causes of cancer-related mortality worldwide, representing a persistent challenge for public health systems [1,2,3]. Although significant advances have been achieved in early diagnosis and therapeutic modalities, including surgery, chemotherapy, radiotherapy, targeted therapies, and immunotherapy, tumor heterogeneity and resistance mechanisms continue to compromise the effectiveness and durability of treatments [4,5].

Within this challenging clinical scenario, the tumor microenvironment (TME) has emerged as a determining factor in neoplastic progression and in the response to oncological therapies [6,7]. This microenvironment consists of a complex and dynamic network of stromal cells, such as cancer-associated fibroblasts (CAFs), immune and endothelial cells, blood and lymphatic vessels, in addition to the extracellular matrix (ECM), which acts as both a structural and functional support for tumor cells [8,9]. In the field of pharmaceutical sciences and tissue engineering, understanding this architecture is crucial for developing bio-inspired models and advanced drug delivery systems.

The ECM, in particular, plays a fundamental role in the regulation of cellular signaling, acting as a reservoir of cytokines and growth factors, while transmitting biochemical and biomechanical stimuli that directly affect cellular proliferation, differentiation, migration, and survival [10]. Among its components, collagen stands out as the most abundant fibrous protein in mammalian tissues, functioning as a natural 3D scaffold that exerts multiple functions in the tumor biology of breast cancer [11].

Alterations in the deposition, organization, and crosslinking of collagen fibers contribute to increased tissue stiffness and promote ECM alignment, favoring collective cell migration and tumor invasion [12,13,14]. These processes are not merely structural: they create a pro-tumoral microenvironment that intensifies uncontrolled proliferation, facilitates metastatic dissemination, and compromises therapeutic success [15,16]. Furthermore, the dense architecture of collagen presents a significant challenge for the diffusion of therapeutic agents, acting as a steric barrier that limits pharmacological efficacy.

Furthermore, the density and organizational pattern of collagen may constitute a physical barrier to drug penetration, also influencing signaling pathways that promote cellular resistance [17,18,19]. A deep understanding of the interface among collagen, tumor cells, and immune cells is essential to optimize therapeutic strategies, particularly in the design of next-generation delivery platforms capable of bypassing these fibrotic barriers [6].

Although previously published review studies have addressed general aspects of the extracellular matrix (ECM) in oncology or the role of collagen in specific tumor-related processes, a gap remains in the literature regarding a critical and integrated analysis of recent evidence correlating structural and functional modifications of collagen with tumor progression and therapeutic response in mammary adenocarcinoma. There is an emerging need to integrate findings on the different collagen subtypes involved with their direct impact on the pharmacokinetics and penetration of chemotherapeutic and immunotherapeutic agents [20,21]. Moreover, these biological insights provide the blueprint for 3D bioprinting technologies aimed at mimicking the tumoral stroma for drug screening.

Additionally, it is relevant to consider that cancer is a biologically dynamic and evolutionary condition, characterized by progressive genomic, epigenetic, and phenotypic changes that remodel the tumor microenvironment and extracellular matrix over time and under therapeutic pressure [22,23]. This plasticity clearly evidences that an exclusive focus on the tumor cell is insufficient to reverse the course of the disease. Variations in survival rates across different global regions, where access to early diagnosis and modern treatments remains disparate, underscore the urgent necessity of identifying universal prognostic factors and therapeutic targets that are intrinsic to tumor biology. In this context, focusing on collagen emerges as a critical area for therapeutic innovation [5].

Therefore, the present review aims to critically consolidate and analyze the most recent scientific evidence regarding the role of collagen as a modulator of tumor progression and therapeutic response in breast adenocarcinoma. By integrating data on collagen dynamics within the TME, this work seeks to identify prognostic and predictive biomarkers, as well as to validate potential therapeutic targets capable of overcoming resistance and optimizing the delivery and distribution of antineoplastic agents through the dense 3D tumor matrix.

## 2. Methods

### 2.1. Study Design and Research Question

This work constitutes a Comprehensive Narrative Review, meticulously designed to systematically and profoundly synthesize the existing evidence concerning the role of collagen in the progression and therapeutic response of breast adenocarcinoma. The central question guiding the data collection and analysis was: “What is the consolidated evidence regarding the role of collagen (dynamics, stiffness, alignment, or metabolism) as a prognostic factor, resistance mechanism, a barrier to drug delivery, and/or therapeutic target in Breast Adenocarcinoma?”

### 2.2. Search Strategy and Data Sources

The electronic search was finalized in November 2025 and encompassed recognized bibliographic databases, including PubMed/MEDLINE, Scopus, and Web of Science (WoS), to ensure maximum scientific and multidisciplinary coverage (bridging molecular biology, oncology, pharmacology, and bioengineering). Additionally, Gray Literature (websites of governmental organizations such as INCA and NCI) was consulted for up-to-date epidemiological data and reports.

#### 2.2.1. Search Terms and Boolean Operators

To optimize the search sensitivity and comprehensively cover all investigation angles, controlled vocabulary terms (MeSH/DeCS) and free-text terms were employed. These were grouped into three conceptual axes and combined using the Boolean operators AND and OR: Collagen/ECM: “Collagen” OR “Extracellular Matrix” OR “Tumor Stroma” OR “Fibrosis” OR “Matrix Stiffness” OR “TACS” OR “LOX” OR “Lysyl Oxidase”; Neoplasm: “Breast Cancer” OR “Breast Neoplasm” OR “Breast Adenocarcinoma” OR “Tumor Microenvironment” OR “TME”; Outcome/Target: “Therapeutic Resistance” OR “Drug Resistance” OR “Prognosis” OR “Metastasis” OR “Target” OR “Biomarker” OR “Drug Delivery” OR “Pharmacological Penetration”.

#### 2.2.2. Publication Period

The search period was strictly defined between January 2011 and November 2025. This temporal window was strategically selected to focus on the most recent literature (the past 15 years), which has seen the consolidation of knowledge regarding the biomechanical role of the matrix.

Classic or seminal articles crucial for establishing the fundamental concepts of tissue stiffness and collagen alignment, published prior to 2011, were included through a manual reference-based search (from the reference lists of the most recent articles) to provide adequate theoretical and historical context.

### 2.3. Eligibility and Study Selection Criteria

The eligibility criteria were rigorously applied in two distinct phases (title/abstract screening and full-text review) and are detailed as follows:

#### 2.3.1. Inclusion Criteria

Study Type: Original studies (in vivo or in vitro), clinical trials, systematic reviews, and meta-analyses.

Topic/Intervention: Articles investigating, primarily or secondarily, the relationship between collagen (any subtype, synthesis, stiffness, alignment, TACS, metabolism, or enzymes like LOX) and breast adenocarcinoma (any histological/molecular subtype), and/or its correlation with therapeutic response (chemotherapy, radiotherapy, or immunotherapy) and studies focusing on the impact of collagen-rich matrices on the diffusion and delivery of antineoplastic agents.

Language: Articles published in the English language.

#### 2.3.2. Exclusion Criteria

Publication Type: Editorials, letters to the editor, conference abstracts, and non-peer-reviewed preprints.

Topic Focus: Studies primarily focused on other ECM components (e.g., elastin, hyaluronic acid, glycosaminoglycans) without emphasis on collagen. Studies focused on other primary cancer sites (e.g., lung, prostate) without direct and explicit translational applicability to the mammary microenvironment.

Access: Full-text articles inaccessible after reasonable acquisition efforts.

## 3. 3D Collagen Network: From Structure to Therapeutic Challenge

The complexity of the 3D collagen network in breast adenocarcinoma requires sophisticated experimental approaches to evaluate its role in tumor progression and drug resistance. Understanding the biomechanical properties of the extracellular matrix (ECM) necessitates models that accurately replicate the desmoplastic reaction. In this context, several experimental platforms, ranging from 2D cultures to advanced 3D bioprinting and in vivo systems, have been developed to study how collagen fiber alignment and density impact therapeutic outcomes [24,25].

### 3.1. In Vitro Models: Recapitulating the 3D Collagen Architecture

While two-dimensional (2D) cell cultures have historically provided insights into cancer cell proliferation and migration, they are fundamentally limited by their inability to replicate the complex 3D collagen topography of the breast tumor microenvironment (TME). In 2D systems, the absence of a structured ECM leads to non-physiological cell morphology and altered gene expression, often resulting in overestimating drug efficacy [26,27].

To overcome these limitations, 3D organoids and scaffolds have emerged as superior platforms for studying the desmoplastic reaction. These models allow for the controlled manipulation of collagen density and stiffness, enabling researchers to investigate how fiber alignment promotes the Epithelial–Mesenchymal Transition (EMT) and physical resistance to drug penetration [28,29,30]. Furthermore, tumor-on-a-chip platforms integrate microfluidic flow to simulate interstitial fluid pressure (IFP), a critical factor in breast adenocarcinoma, where a dense collagen network hinders the convective transport of nanotherapeutics [31,32].

### 3.2. In Vivo Models: Biomechanical Remodeling and Host Interactions

In vivo systems remain the gold standard for evaluating the dynamic remodeling of the collagen matrix. Immunosuppressed mice (Xenografts) are instrumental in studying human-derived breast cancer cells; however, the murine host ECM may not fully recapitulate the cross-linking patterns found in human desmoplasia [33,34,35].

In contrast, Genetically Modified Models (GMMs), particularly those utilizing CRISPR/Cas9, allow for the precise deletion or overexpression of collagen-modifying enzymes, such as Lysyl Oxidase (LOX). These models are vital for demonstrating how collagen “stiffening” feedback loops drive tumor progression and immune evasion in a physiologically relevant context [36,37]. Spontaneous and syngeneic models (e.g., BALB/c) offer the advantage of an intact immune system, which is essential for investigating how the 3D collagen shield protects tumor cells from T-cell infiltration, a major hurdle in current immunotherapy [38,39,40].

### 3.3. The Ehrlich Adenocarcinoma (EAC) Model in Collagen Research

The Ehrlich Adenocarcinoma (EAC) model, specifically in its Solid Ehrlich Tumor (SET) form, serves as a robust preclinical platform for studying breast-like malignancies. Although EAC is highly aggressive and pleomorphic, its value in collagen research lies in its rapid induction of a stromal response upon subcutaneous inoculation [41,42,43].

While the EAC model lacks a highly metastatic phenotype, it provides a reproducible environment to test ECM-modulating agents and natural compounds aimed at reducing collagen density. By monitoring changes in the tumor’s physical consistency and stromal architecture, the EAC model facilitates the screening of therapies designed to “soften” the TME, thereby improving the delivery of conventional cytotoxic agents [44,45,46].

### 3.4. The 3D Collagen Network: Architectural Determinant of the Breast TME

In breast adenocarcinoma, the Extracellular Matrix (ECM) transcends its role as a structural scaffold, acting as a dynamic regulator of tumor evolution. While the ECM comprises proteoglycans (PGs), glycosaminoglycans (GAGs), and elastin, collagen is the primary driver of the physical barriers encountered during drug delivery [47,48].

### 3.5. Specific Role of Collagen in Tumor Progression and Immune Response

#### 3.5.1. Structural Hierarchy as a Mechanical Determinant

The collagen superfamily, particularly the fibril-forming types I, II, and III, provides the essential structural scaffold of the mammary gland. Type I collagen, representing 90% of the total content, is organized into a triple-helix structure (tropocollagen) that assembles into microfibrils and fibers [49,50]. In the context of adenocarcinoma, this hierarchical organization is hijacked. The repetitive Gly-X-Y triplets and the dense interchain hydrogen bonding provide the mechanical stability that, when dysregulated by Cancer-Associated Fibroblasts (CAFs), results in the characteristic stiffness of the breast tumor microenvironment (TME) [51,52].

#### 3.5.2. From Native to Hydrolyzed Collagen: Pharmaceutical Relevance

The transition from native to hydrolyzed collagen—via enzymatic or thermal denaturation—alters its physicochemical properties, increasing solubility and bioavailability [53,54]. While native collagen maintains the triple-helix integrity necessary for structural studies, hydrolyzed peptides and collagen-derived hydrogels have emerged as versatile biomaterials. In oncology, these hydrogels are increasingly used in 3D bioprinting and drug delivery systems, as they mimic the hydrated environment of the ECM while allowing for the controlled release of therapeutic agents and the scavenging of free radicals [55,56].

#### 3.5.3. Collagen as a Functional Driver of Progression and Biomarker

As synthesized in Table 1, the remodeling of collagen expression is a hallmark of breast adenocarcinoma. Type I collagen (COL1A1) stands out as the primary modulator of the ECM, where its overdeposition is directly coupled with LOX-mediated crosslinking and matrix stiffening [57,58]. This “architectural plasticity” is not merely structural; it actively facilitates cellular trafficking. Imaging studies reveal that migratory tumor cells utilize linearized collagen fibers as “tracks” to escape the primary tumor, a process guided by Tumor-Associated Collagen Signatures (TACS), particularly the perpendicular alignment known as TACS-3 [59,60,61].

Beyond Type I, Table 2 highlights the role of less explored variants like collagens III, VI, and XII. For instance, COL12A1 secreted by CAFs can alter the organization of Type I fibers, creating a pro-metastatic niche [65]. These structural shifts are now recognized as robust prognostic biomarkers (Table 3), where high stromal Type IV expression and intratumoral collagen uniformity independently predict reduced recurrence-free survival [61,66].

#### 3.5.4. Mechanotransduction and the “Immune Desert”

The interaction between tumor cells and the collagen matrix is mediated by specialized receptors, including integrins, DDR1, and DDR2 [74]. As summarized in Table 4, this biomechanical signaling activates the FAK/YAP and PI3K/AKT/mTOR pathways, leading to the formation of invadopodia—actin-rich protrusions that degrade the ECM and accelerate invasion [75,76].

A critical pharmaceutical challenge identified in recent studies is the role of collagen in immune reprogramming. The densely compacted and aligned collagen environment acts as a “physical shield” or immune desert, which:

- Excludes cytotoxic cells by limiting the infiltration of CD8+ T lymphocytes and NK cells [76].
- Polarizes macrophages by triggering the transition of Tumor-Associated Macrophages (TAMs) toward an immunosuppressive M2 phenotype [79,81].
- Hinders immunotherapy by creating a barrier that prevents contact between immune checkpoint inhibitors and their cellular targets.

#### 3.5.5. Impact of Collagen Isoforms on Clinical Outcomes

The functional duality of collagen is evidenced in Table 5. While COL1A1 is universally associated with poor prognosis and hormonal therapy resistance [68,70], some isoforms like COL3A1 may exert a “tumor-restraining” effect depending on the Type I/Type III ratio [82]. Furthermore, hypoxia in the TME exacerbates this complexity by upregulating LOX and MMPs, which further stiffen the matrix and promote EMT [3,74].

Experimental strategies to modulate this “mechanobiology”—such as copper depletion with tetrathiomolybdate (TM) or LOX inhibition—are currently being explored to “soften” the TME and restore drug sensitivity [57,70].

### 3.6. Therapeutic Approaches and Translational Innovation

#### 3.6.1. From Conventional Cytotoxicity to Matrix-Targeting Strategies

Conventional chemotherapy and radiotherapy, while effective, often fail in the context of highly desmoplastic breast tumors due to poor drug penetration. The dense collagenous stroma creates a high Interstitial Fluid Pressure (IFP), which counteracts the convective transport of small molecules and nanocarriers alike [42,88]. Modern oncology is shifting towards Targeted Therapies and Immunotherapy (e.g., anti-PD-1/PD-L1); however, their efficacy is strictly dependent on the ability to bypass the “collagen shield” that characterizes the immune desert [89,90].

#### 3.6.2. Pharmacological Modulation of the Collagen Matrix

The mechanical properties of the TME, governed by Type I, III, and IV collagens, are now viewed as druggable targets.

Stiffness Normalization: Strategies aimed at inhibiting Lysyl Oxidase (LOX) or reducing fiber cross-linking are being explored to “soften” the TME, thereby lowering IFP and enhancing the delivery of co-administered chemotherapeutics [91].

The Dual Role of Collagen: While COL1A1 promotes invasion through TACS-3 alignment, recent evidence suggests that Type III Collagen (Col3) may act as a tumor-suppressive modulator. Recombinant human Col3 (rhCol3) hydrogels have shown potential in reducing tumor growth and metastatic burden, offering a biocompatible route for stromal “renormalization” [92].

#### 3.6.3. Exosomes as Bioactive Shuttles in the Remodeled ECM

Exosomes (40–160 nm) act as the primary communication axis between tumor cells and the ECM. In a rigid environment, cancer-derived exosomes carry a specialized cargo, which includes MMP-14, TGF-β, and STAT3, that triggers the differentiation of resident fibroblasts into Cancer-Associated Fibroblasts (CAFs) [93,94]. From a biopharmaceutical standpoint, these vesicles represent a double-edged sword: they drive the pre-metastatic niche formation but also serve as inspiration for biomimetic nanocarriers. Understanding how the collagen matrix alters exosomal tropism is crucial for designing the next generation of targeted drug delivery systems [49,95].

## 4. Conclusions and Translational Implications

This review systematically consolidates the evidence positioning collagen as a central, bioactive regulator of breast adenocarcinoma. The transition from a 2D understanding of tumor biology to a 3D mechanobiological paradigm is essential for overcoming therapeutic resistance. We draw the following conclusions:

Biomechanical Barriers: Collagen architecture (TACS-3) and LOX-mediated stiffening are the primary physical obstacles to drug extravasation and immune cell infiltration.

Diagnostic Value: Collagen signatures should be integrated into clinical practice as prognostic biomarkers to stratify patients who may benefit from matrix-priming therapies.

Future Research: The pharmaceutical industry must prioritize the development of “Stromal-Permeable” nanotechnologies and inhibitors of collagen cross-linking.

By shifting the therapeutic focus from the tumor cell alone to the 3D collagenous architecture, we pave the way for more effective, personalized, and “stroma-aware” treatments for breast cancer.

## Figures and Tables

**Table 1:** Functional roles and alterations of collagen types in the progression and therapeutic response of breast adenocarcinoma.

Study	Collagen Modification	Signaling Changes	Progression Effects	Clinical Implications	References
Levental et al., 2009	No mention found	No mention found	Collagen crosslinking present, drives ECM stiffening	ECM stiffening associated with crosslinking	[57]
Jena and Janjanam, 2018	No mention found	No mention found	Lysyl oxidase-like 2/4 (LOXL2/LOXL4) catalyze cross-linking	Hardened ECM, increased tumor stiffness	[62]
Fernández-Nogueira et al., 2021	High collagen deposition in TME	Perpendicular orientation in Basal-like/HER2 ductal carcinoma in situ	No mention found	Matrix stiffness increases, linked to therapy resistance	[58]
Martínez and Smith, 2021	High proportion of fibrillar collagens	Dense structure, high fibrillar content	Lysyl oxidase (LOX) catalyzes cross-linking	Tumor periphery ~7x stiffer than interior; rigidity 0.8 → 4.0 kilopascals	[63]
Lepucki et al., 2022	Increased collagen deposition, stromal stiffness	Dense network perpendicular to tumor border	LOX pathway produces mature crosslinks	Increased stiffness due to crosslinking and deposition	[64]
Papanicolaou et al., 2022	Upregulated in late-stage tumors	Increased bundle width and linearity	Collagen XII stabilizes collagen I fibrils, regulates 3D organization	Tumor stiffness increases in late stages; reduced with collagen XII depletion	[65]

**Table 2:** Effects of collagen on tumor progression: structural modifications and mechanical impacts.

Study	Collagen Modification	Signaling Changes	Progression Effects	Clinical Implications	References
Iyengar et al., 2005	Collagen VI from adipocytes	NG2/AKT/beta-catenin/cyclin D1	Promotes early hyperplasia, tumor growth	Collagen VI as early progression target	[67]
Levental et al., 2009	Collagen crosslinking, ECM stiffening	Integrin/PI3K signaling	Promotes invasion, increases tumor incidence	LOX inhibition reduces malignancy	[57]
Acerbi et al., 2015	Collagen I deposition/linearization, ECM stiffening	TGF signaling, macrophage infiltration	Aggressive subtypes show more stiffening, immune infiltration	Stiffness/linearization as aggression markers	[13]
Liu et al., 2018	COL1A1 expression	CXCR4 signaling	Promotes metastasis, poor survival (estrogen receptor-positive)	COL1A1 as prognostic/therapeutic target	[68]
Shea et al., 2018	High collagen I density (desmoplasia)	AKT-mTOR, YAP activation	Increased cancer stem cells, more/larger lung metástases	mTOR inhibition effective in primary, not metastatic, tumors	[69]
Jallow et al., 2019	COL1A1-dense ECM	Increases AP-1 activity	Tamoxifen-stimulated proliferation/metastasis	ECM context alters therapy response	[70]
Byrne et al., 2021	Collagen I-rich ECM	Alters senescence pathway transcription	No proliferation increase; ECM sensitizes estrogen receptor-negative cells to therapy	Suggests the need for complex ECM in models	[71]
Liu et al., 2022	TM regimes crosslinkling, ECM stiffening	T-cell migration/activation, less myeloid-derived suppressor cells	Decreased metastasis, increased T-cell infiltration	TM improves survival, especially in triple-negative breast cancer	[72]
Papanicolaou et al., 2022	Collagen XII organizes collagen I, increases stiffness	Myofibroblast phenotype, matrix remodeling	Promotes invasion, metastasis; increased bundle width/density	Collagen XII predicts poor survival, high metastatic risk	[65]
Volk et al., 2011	Type III collagen deficiency leads to a tumor-permissive matrix; Col3:Col1 ratio	No mention found; affects proliferation/apoptosis	Increased proliferation, reduced apoptosis, higher recurrence/metastasis with low type III collagen	High Col3:Col1 ratio predicts improved survival	[73]

**Table 3:** Characteristics of collagen as prognostic biomarkers in breast cancer.

Study	Target	Mechanism	Therapeutic Approach	Clinical Status	References
Iyengar et al., 2005	Collagen VI, NG2 receptor	Promotes early growth via AKT/beta-catenin	Targeting NG2/collagen VI axis	Preclinical	[67]
Levental et al., 2009	LOX, integrin signaling	ECM stiffening, invasion	LOX inhibition, integrin blockade	Preclinical	[57]
Liu et al., 2018	COL1A1	Promotes metastasis, chemoresponse	COL1A1 knockdown	Preclinical/clinical biomarker	[68]
Shea et al., 2018	Collagen I density, mTOR	Cancer stem cell niche, metastasis	mTOR inhibition (rapamycin), Copper depletion	Preclinical	[69]
Jallow et al., 2019	ECM density (COL1A1)	Alters hormone therapy response	Targeting both estrogen receptor and ECM	Preclinical	[70]
Liu et al., 2022	LOXL2, PRO-C3, C6M, C1M	Collagen crosslinking/processing	Copper depletion (tetrathiomolybdate)	Phase II clinical	[31]
Papanicolaou et al., 2022	Collagen XII	Regulates collagen I, promotes metastasis	Knockdown in cancer-associated fibroblasts	Preclinical	[65]
Volk et al., 2011	Type III collagen (Col3)	Tumor-restrictive ECM	Col3 supplementation (hydrogels)	Preclinical	[73]

**Table 4:** Comparative synthesis of collagen-related mechanisms and signaling pathways in cellular populations of the tumor microenvironment.

Study	Cell Populations Studied	Mechanisms/Signaling Pathways	References
Levental et al., 2009	Breast cancer cells	Collagen crosslinking enhances integrin clustering, phosphoinositide 3-kinase (PI3K) signaling, invasion	[57]
Mao et al., 2013	Breast cancer cells, CAFs, TAMs, endothelial cells, adipocytes	CAFs promote invasion, angiogenesis, resistance via ECM/collagen; CAFs activate mitogen-activated protein kinase (MAPK)/protein kinase B (Akt), epidermal growth factor receptor (EGFR)/PI3K/Akt, WNT16B/NF-B pathways	[77]
Oskarsson, 2013	Breast cancer cells	ECM proteins, receptors, and modifiers mediate resistance via signaling pathways	[78]
Jena, Janjanam, 2018	Breast cancer cells, CAFs, TAMs, adipocytes, endothelial cells	Hardened ECM upregulates extracellular signal-regulated kinase 1/2 (ERK1/2), downregulates Janus kinase 2/signal transducer and activator of transcription 5 (JAK2/STAT5); heparanase activity, hepatocyte growth factor (HGF)/c-Met pathway	[62]
Deligne, Midwood, 2021	Macrophages, CAFs, endotelial cells	ECM modulates immune cell infiltration, polarization; matrix molecules influence tumor-associated macrophage (TAM) phenotype	[79]
Fernández-Nogueira et al., 2021	Breast cancer cells, CAFs, fibroblasts	High collagen activates JNK1, stress/inflammatory pathways; CAFs secrete matrix metalloproteinases (MMPs), modify collagen orientation	[58]
Martínez, Smith, 2021	Breast cancer cells, CAFs, macrophages, T cells, myofibroblasts	Collagen crosslinking via LOX, Yes-associated protein/transcriptional coactivator with PDZ-binding motif (YAP/TAZ) pathway, immune exclusion, metabolic reprogramming	[63]
Tamayo-Angorrilla et al., 2022	CAFs (implied), other TME cells	ECM remodeling affects cell behavior and therapy response (mechanisms not specified)	[80]
Lepucki et al., 2022	Breast cancer cells, CAFs, T cells, TAMs, endritic cells, endothelial cells, adipocytes	Collagen crosslinking enhances integrin clustering, phosphoinositide 3-kinase (PI3K) signaling, invasion	[64]
Papanicolaou et al., 2022	Breast cancer cells, cancer-associated fibroblasts (CAFs)	Collagen XII alters collagen I organization, creates pro-invasive microenvironment; ECM stiffness may affect nuclear factor kappa B (NF-B)/c-Jun N-terminal kinase (JNK) pathways	[65]

**Table 5:** Expression patterns and collagen types and their effects on tumor progression.

Study	Collagen Type(s)	Expression Pattern	Impact on Progression	Clinical Correlation	References
Conklin et al., 2011	No mention found (TACS-3)	Bundles of straight, aligned collagen perpendicular to tumor boundary	TACS-3 reported as an independent predictor of poor disease-specific and disease-free survival	TACS-3 reported to correlate with stromal syndecan-1 expression	[16]
Brodsky et al., 2016	Type X collagen alpha 1 chain (COL10A1)	Increased stromal COL10A1 in non-responders	Reported association with poor pathologic response	High COL10A1 and low tumor-infiltrating lymphocytes reported to predict poor neoadjuvant therapy response	[82]
Thangavelu et al., 2016	Collagen type XVII alpha 1 chain (COL17A1)	Underexpression in breast cancer; overexpression in cervical cancer	Underexpression in breast cancer reported to be linked to increased invasion and poor	COL17A1 underexpression reported to predict poor distant metastasis-free, metastasis-free, recurrence-free, and overall survival	[83]
Esbona et al., 2018	No mention found	High local density, alignment, and perpendicular orientation (tumor-associated collagen signature-3, TACS-3)	Reported to predict poor overall survival	High COX-2, CD68, CD163 and aligned collagen, reported to predict worse survival	[60]
Liu et al., 2018	Collagen type I alpha 1 chain (COL1A1)	High expression in breast cancer cells and patients	Reported to promote metastasis; knockdown reported to inhibit metastasis	High COL1A1 reported to be associated with poor survival in estrogen receptor-positive patients	[68]
Ren et al., 2018	Collagen type V alpha 1 chain (COL5A1)	Overexpressed in invasive ductal carcinoma, co-polymerizes with type I collagen	Reported association with increased cell viability, migration, invasion; knockdown reported to reduce these	High COL5A1 mRNA reported to be associated with distant metastasis-free survival; overexpression linked to estrogen/progesterone receptor status	[84]
Tomko et al., 2018	Collagen type XII alpha 1 chain (COL12A1), tenascin C (TNC), thrombospondin-2 (THBS-2)	Aligned collagen fibers (TACS-3) co-localized with TNC, THBS-2	TACS-3 and associated proteins reported to correlate with poor distant metastasis-free survival	COL12A1, TNC, and THBS-2 signature reported to predict poor outcome	[85]
Wang et al., 2019	Type X collagen alpha 1 chain (COL10A1), elastin	COL10A1/elastin complex forms amorphous clumps in tumor stroma	Reported correlation with poor pathologic response to neoadjuvant chemotherapy	High COL10A1/elastin expression reported to correlate with poor outcome	[86]
Wei et al., 2019	Type I collagen, PLOD2	Adipocyte-driven remodeling reported to increase alignment and density at invasive front	Reported to promote metastasis; PAI-1/PLOD2 axis reported to drive collagen reorganization	High PAI-1 reported to correlate with poor prognosis, especially in HER2-negative/estrogen receptor-negative and triple-negative cancers	[87]
Volk et al., 2011	Type III collagen (COL3A1), Type I collagen (COL1A1)	High type III to type I collagen ratio in noninvasive regions; more aligned fibers in invasive areas	Type III collagen reported as tumor-restrictive; high type III to type I collagen ratio reported to improve survival and reduce metastasis	High type III to type I collagen ratio associated with improved overall, disease-free, and progression-free survival	[73]

## Data Availability

No new data were created or analyzed in this study. Data sharing is not applicable to this article.
